# Performance of alternative measures to body mass index in the assessment of moderate and severe under-nutrition among acutely unwell patients hospitalized in a TB ward in the Philippines: A cross-sectional study

**DOI:** 10.1371/journal.pone.0215968

**Published:** 2019-05-16

**Authors:** Laura V. White, Nathaniel Lee, Flora P. Marin, Naomi R. Saludar, Tansy Edwards, Sharon E. Cox

**Affiliations:** 1 Nagasaki University, School of Tropical Medicine and Global Health, Nagasaki, Japan; 2 Royal Free Hospital, London, United Kingdom; 3 San Lazaro Hospital, Manila, Philippines; 4 Tropical Epidemiology Group, Faculty of Population Health, London School of Hygiene and Tropical Medicine, London, United Kingdom; 5 Faculty of Population Health, London School of Hygiene and Tropical Medicine, London, United Kingdom; University of Mississippi Medical Center, UNITED STATES

## Abstract

Body mass index (BMI) kg/m^2^ is a key screening tool for under-nutrition in adults, but difficult to obtain in immobile or unwell patients, particuarly in low resource settings, due to inability to accurately measure both weight and height. Mid-upper arm circumference (MUAC) is used to assess under-nutrition in children under 5 years but no standardised cut-off values exist for adults. In a cohort of adult Filipino patients admitted to a tuberculosis ward we assessed (i) cut-offs for MUAC to predict moderate under-nutrition (BMI <17kg/m^2^), (ii) the performance of limb lengths to predict height and; (iii) associations of body fat percentage from skinfolds and hand grip-strength with BMI. In 303 patients with MUAC and BMI at admission, aged 18–80 years (mean = 45.5, SD:14.8), BMI ranged from 11.2–30.6 kg/m^2^ and 141 (46.5%) had BMI <17.0 kg/m^2^. Using receiver operator curves, MUAC cut-offs were identified as <20.5cm for males (sensitivity: 89%, specificity: 84%) and <18.5cm for females (sensitivity: 91%, specificity: 89%), for BMI<17.0 kg/m^2^. Using published equations, knee height had the lowest mean difference between predicted and measured heights compared to ulnar or demi-span: (-0.98 cm, 95% CI: -1.51/-0.44). Both grip-strength and body fat percentage were positively associated with BMI, in separate linear regression models with exposure-age-sex interactions (adjusted-R-squared values: 0.15, 0.66, respectively). MUAC can predict moderate acute under-nutrition with high positive predictive value. Further research is required to determine the performance of alternative measures to BMI to predict mortality or adverse outcomes in acutely unwell patients.

## Introduction

Under-nutrition is a common risk factor for mortality among hospitalized patients [1] in both low and high resource settings. Under-nutrition is also associated with prolonged length of stay, increased likelihood of re-admission [2] and higher risk of infections [3]. Early diagnosis and intervention for under-nutrition can result in reduced costs, morbidity and mortality [4, 5]. Therefore, screening for under-nutrition on admission, to identify patients at risk and those who require nutrition interventions should occur for all patients. Several ‘nutrition scores’ such as the subjective global assessment (SGA) and the malnutrition universal screening tool ‘MUST’ exist but rely on body mass index (BMI) to determine accurate risk classification [6]. Although BMI is widely used in nutrition screening, obtaining accurate weight or height measurements among severely unwell or immobile inpatients is often not feasible. Therefore, the use of BMI as an indicator is a barrier to identification of under-nutrition in acutely unwell or immobile patients. Some tools like the MUST provide alternative measures for patients where height, or height and weight cannot be obtained, such as the use of limb length and predictive equations to determine height and the use of mid-upper arm circumference (MUAC) in the absence of weight [7]. However, the majority of these predictive equations have been generated using Caucasian participants and studies have demonstrated these equations may not predict height as accurately in other ethnicities [8, 9]. For immobile patients, limb length predictive equations still require the estimation or self-reporting of an accurate weight to determine BMI and in many hospitalized patients recent weight loss renders their ‘usual weight’ inaccurate. When both weight and height are unavailable, alternative measures such as MUAC have been investigated in place of BMI [10]. However, despite a systematic review and individual participant data meta-analysis on the possible MUAC cut-off to identify under-nutrition, as defined by the WHO cut off of <18.5 kg/m^2^, [11] there are still no internationally recognized cut-off values for MUAC to define under-nutrition in adults.

Under-nutrition increases the risk of developing active tuberculosis (TB) disease [12]. TB can also directly and indirectly cause under-nutrition. This can happen through metabolic changes which can result in clinical wasting [13], alongside with changes in appetite, ability to eat, anti-tuberculosis treatment side effects and impacts of the disease and treatment on household incomes [14]. For individuals hospitalized with TB the risk of poor clinical outcomes, associated with under-nutrition, is particularly high, especially TB related mortality [15, 16]. In low income countries especially, patients may present to hospital settings with clinically advanced disease resulting in a high prevalence of TB associated wasting and mortality [15]. The WHO recommends additional nutritional support should be provided for these patients [17], although currently there is lack of evidence to support what kinds of interventions and of efficacy on reducing mortality or on TB treatment-related outcomes. In these settings BMI may be a barrier to screening for under-nutrition and identifying patients in need of nutrition interventions. While a BMI<18.5kg/m^2^ signifies underweight according to WHO criteria, under-nutrition can be further subdivided into severity categories of 17–18.49 (mild thinness), 16–16.99 (moderate thinness) and <16 (severe thinness) [18]. Severity of under-nutrition in TB patients, especially in limited resource settings will determine if and what nutritional interventions are to be allocated [17, 19]. In order to improve the screening and accuracy of under-nutrition diagnosis in TB patients (inpatient and outpatient) and their subsequent management, simpler anthropometric measures or more functional measures of under-nutrition than BMI may be more appropriate.

The objectives of this study were to investigate the feasibility and accuracy of predicting BMI in severely unwell or immobile hospitalised patients, using admission data from the TB ward at San Lazaro Hospital in Manila, Philippines. Of particular interest, was to determine whether MUAC, as a simple measure possible in all patients regardless of mobility, can predict BMI and moderate or severe under-nutrition assessed by BMI of <17.0 kg/m^2^.

## Materials and methods

### Study design and setting

A cross-sectional study of admission data, collected as part of a prospective cohort study in the TB ward of San Lazaro Hospital; a large, public infectious disease hospital serving Metro Manila in the Philippines.

#### Study participants, recruitment, inclusion and exclusion criteria, and enrolment procedures

**Fig 1:**
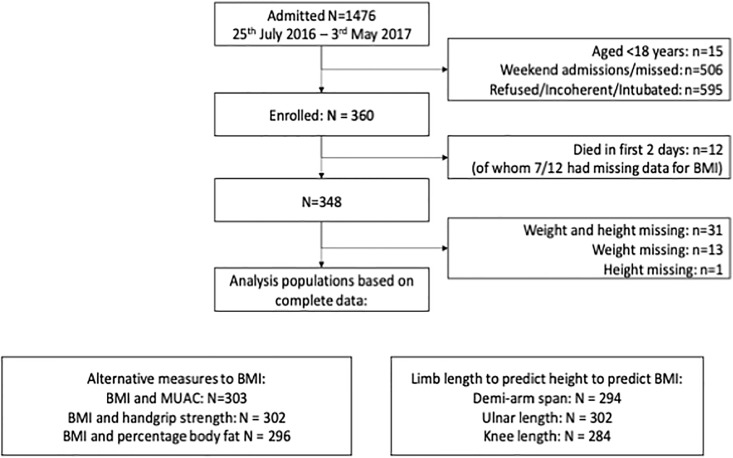
Participants. MUAC = mid-upper arm circumference, BMI = body mass index (kg/m^2^).

All patients aged at least 18 years old admitted to the TB ward at San Lazaro Hospital, Manila were eligible for enrolment with the exception of patients who were unable to give written consent due to incapacity, (unconscious/incoherent or too acutely unwell to participate such as severe shortness of breath) who were excluded (Fig 1). Study nurses checked the ward admissions register twice daily on weekdays only and requested consent to participate from individuals within 24 hours of admission. Participants were enrolled from July 2016–May 2017.

### Data collection

#### Anthropometry

Trained research nurses (3) demmonstrated inter-individual differences for MUAC, height, limb length and skin-fold measurements of ≤0.5 cm before data collection. Research nurses completed anthropometric measurements within 24 hours of admission, after patients were stabilised as necessary. Due to clinical status or immobility, not all anthropometric measurements could be taken on all patients. When measurement of height was not possible at admission, height was measured at discharge. Weights were taken (to the nearest 0.1kg; Seca 803 Clara Digital Personal Non-Medical Scale) on a flat surface with the patient standing upright and unassisted without shoes. Heights were taken (to nearest 0.5cm; Seca 216 Mechanical Stadiometer) without shoes or socks with the patient standing unsupported and positioned fully upright with the lower border of the left orbit and the upper margin of the external auditory meatus horizontal. MUAC was taken (to the nearest 0.5cm; Seca 201 measuring tape) at the midpoint between the acromial process and olecranon on the posterior aspect of the study participant with shoulders adducted and olecranon flexed to 90 degrees. Limb length measurements were taken to the nearest 0.5cm using a Seca 201 measuring tape. Ulna limb length measurements were measured between the point of the olecranon process and the midpoint of the styloid process. Knee height was obtained with the knee and ankle bent to a 90 degree angle while patient lied supine, measuring from under the heel of the foot to the anterior surface of the thigh (~3cm above patella) and ensuring the measuring tape was in line with and parallel to the tibia and is over the lateral malleolus. Demi-arm span was measured from the midpoint of the sternal notch to the web between the middle and ring finger, with the arm outstretched in a horizontal position in line with the shoulders ensuring the arm was flat and the wrist was straight. Limb lengths and MUAC were taken on both sides of the body in duplicate; if measurements on a single side differed by greater than 1 cm, a third measurement was taken and the average of the two closest results was recorded. Grip strength was taken with the participants arm bent to 90 degrees and their elbow supported by the research nurse (kg; Jamar Hydraulic Hand Dynamometer Lafayette Instruments, USA). Grip measurements were taken alternatively on the left and right arms three times and the highest value for both arms was recorded in kg. Skinfold thickness were measured to the nearest 1mm on the right side of the body at four sites (tricep, bicep, subscapular and suprailiac) in accordance with standard procedures using Harpenden calipers model 68875, Country Technology [20]. Measurements were taken twice, the mean was used if within 1mm, otherwise, a third measurement was taken and the mean of the two closest measurements used. The mean of two MUAC measurements from the patient’s non-dominant arm was analysed. Grip strength was analysed as the highest value from three measurements from each hand. Calculations of proportion of percentage body fat used the equations of Durnin and Womersley [21] (S1 File).

### Statistical methods

Data were entered directly into Open Data Kit 2.0 [22] software by research nurses and uploaded to a secure server daily. Data were analysed using Stata, version 14.1 (College Station TX: StataCorp LP).

The primary analysis was to demonstrate the association between MUAC and BMI and cut-off values for MUAC best corresponding to a BMI classification of under-nutrition (BMI<17 kg/m^2^). The sample size was fixed; determined by the sample size of a cohort study of inpatients to study mortality in 348 patients who met inclusion criteria and survived to day three of admission. According to Machin *et al* (2009), 62 patients should allow estimation of specificity and sensitivity of 80–95% with absolute precision of ±10%, therefore if the sample needs to be stratified by age or sex, precision should still be adequate [23].

Secondary analyses included i) investigations of associations between BMI and percentage body fat as another measure of nutritional status, and handgrip strength as a measure of “functional” nutritional status [24], so as to assess the potential usefulness of these as alternatives to BMI when height and weight cannot always be measured, and ii) investigate predictors of height from different limb length measures in order to impute height data to obtain BMI values where weight but not height is available.

After visual inspection for normality, continuous data were summarised using mean (standard deviation, SD) and categorical data as raw data with percentages.

#### Relationship between MUAC and BMI and identification of MUAC cut-off values for malnutrition

Linear regression was used to model the relationship between MUAC and BMI as continuous variables. It was hypothesised that the relationship may be curvilinear and vary by sex and age group (21–40, 41–66, >65 years). Strong interactions would suggest a need for stratified cut-off values, in subsequent primary analysis to identify cut-off values for MUAC to predict undernutrition as BMI<17kg/m^2^. First, the strength of evidence for a linear or quadratic relationship was assessed by comparing a model with a linear and quadratic term to a model with a linear term for MUAC using a likelihood ratio test (LRT). The pre-specified retention criterion was p<0.1. Next, age group and sex were added to the model with either a linear or quadratic relationship between MUAC and BMI. Then evidence of i) a MUAC-sex interaction after adjustment for age group, ii) a MUAC-age group interaction after adjustment for sex and iii) a MUAC-sex-age interaction was assessed using LRTs. Interactions in i) and ii) were tested in comparison to the model adjusting for age and sex and the interaction in iii) was tested against a model with a significant two-way interaction. The relationship between MUAC and BMI is presented graphically based on results from the regression modelling, demonstrating either a linear or quadratic relationship, with stratification where interactions were significant.

Moderate under-nutrition was defined as BMI<17 kg/m^2^ and severe under-nutrition as BMI<16 kg/m^2^. Cut-off values for MUAC to identify moderate under-nutrition were evaluated in increments of 0.5 cm against moderate under-nutrition defined as BMI<17 kg/m^2^. Stratifying by sex; sensitivity, specificity, positive predictive value, negative predicted values and false negative rate with corresponding 95% CIs were calculated for each cut-off value, along with the area under the curve (AUC, with 95% CIs) from a non-parametric receiver operating curve (ROC) analysis. A MUAC cut-off to diagnose moderate under-nutrition was selected, by sex, based on maximizing sensitivity to reduce missed cases (false negative rate (FNR) = 1 –sensitivity) while maintaining a specificity of at least 80%. This process was repeated to identify severe under-nutrition defined as BMI<16 kg/m^2^. The STARD checklist is available in S2 File.

#### Relationship between handgrip strength and BMI and body fat and BMI

Linear regression was used to model the relationship between handgrip strength and BMI and between body fat and BMI, as continuous variables, using the same approach as described above for MUAC and BMI.

#### Limb length as a predictor of height and using predicted height values to calculate BMI

Height was predicted from each of the three limb measurements; demi-span length and knee height from a published equation from a Filipino population [25] and ulnar length from a published equation from Vietnam [26]. Predicted height was plotted against measured height. The difference between predicted and measured height for each limb length was plotted against the mean of the predicted and measured height [27]. The mean differences (95% CIs) between predicted and measured heights were calculated as an indication of limits of agreement [27]. Predicted heights were used to re-calculate BMI for patients with weights available. The sensitivity, specificity, positive predictive value, negative predictive value and false negative value (1-sensitivity) of BMI categorisation based on predicted heights was compared to categorisation using BMI based on measured values.

### Ethical considerations

Research was conducted according to the declaration of Helsinki. Ethical approval was obtained from Nagasaki University Tropical Medicine Institute (NEKKEN) and San Lazaro Hospital Research Ethics and Review Unit. Informed written consent was obtained from the participants prior to data collection.

## Results

### Participants

BMI was available for 303 (87%) of 348 enrolled patients who survived to day 3 of admission (Fig 1) of whom 69.5% were male and 9.2% were aged more than 65 years (Table 1). BMI ranged from 11.2–30.6 kg/m^2^ and 46.5% (141/303) had moderate or severe under-nutrition with a BMI<17 kg/m^2^ and 35.9% severe under nutrition with a BMI<16.0 kg/m^2^ (103/303), whilst 5.0% (15/303) were overweight (BMI>25 ≤30 kg/m^2^) and only 1 patient obese (BMI >30 kg/m^2^). There was no difference in the proportion of those with moderate/severe under-nutrition between females (45/92 [48.9%]) and males (96/211 [45.5%]). The prevalence of clinical conditions that may associate with body composition was 15% for diabetes and 6.3% for HIV, but 60.3% had unknown HIV status (Table 1) due to refusal of testing. Presence of oedema was not recorded for any of the admissions. There were no apparent differences in the distribution of age, sex or assessed anthropometric measurements between those with and without BMI data.

**Table 1:** Demographic, clinical and anthropometric characteristics of patients. ^*1*^ Diabetes defined as HbA1c ≥ 6.55 or currently on diabetes medication.

		All patients (N = 348)		BMI Available (N = 303)	
	Category, summary measure	N	summary	N	summary
**Sex**	Female n (%)	348	106 (30.5%)	303	92 (30.4%)
**Age distribution in women**	Aged 18–40 years, n (%)	106	36 (34.0)	92	29 (31.5)
	Aged 41–65, years n (%)		57 (53.8)		53 (57.6)
	Aged >65, years n (%)		13 (12.3)		10 (10.9)
**Age distribution in men**	Aged 18–40 years, n (%)	242	103 (42.6)	211	89 (42.2)
	Aged 41–65 years, n (%)		120 (50.0)		104 (49.3)
	Aged >65 years, n (%)		19 (7.9)		18 (8.5)
**Diabetic**^**1**^	n (%)	344	53 (15.4)	301	47 (15.6)
**HIV status**	Negative, n (%)	348	116 (33.3)	303	112 (37.0)
	Positive, n (%)		22 (6.3)		17 (5.6)
	Unknown, n (%)		210 (60.3)		174 (57.4)
**Food intake last month**	No decrease, n (%)	228	99 (43.4)	211	93 (44.1)
	Moderate decrease, n (%)		112 (49.1)		104 (49.3)
	Severe decrease, n (%)		17 (7.5)		14 (6.6)
**Age (years)**	mean (SD)	348	45.3 (15.0)	303	45.5 (14.8)
**Height (cm)**	mean (SD)	316	158.6 (8.3)	303	158.9 (8.1)
**BMI (kg/m**^**2**^**)**	mean (SD)	303	17.9 (3.7)	303	17.9 (3.7)
**Ulnar length (cm)**	mean (SD)	347	24.4 (1.7)	302	24.4 (1.7)
**Knee height (cm)**	mean (SD)	321	47.8 (3.3)	284	48.0 (3.3)
**Demi-span length (cm)**	mean (SD)	338	74.4 (4.7)	294	74.6 (4.7)
**MUAC (cm)**	mean (SD)	348	20.0 (3.6)	303	20.3 (3.5)
**Handgrip strength (kg)**	mean (SD)	346	18.8 (10.5)	302	20.0 (10.2)
**Body fat (%)**	mean (SD)	335	17.5 (8.1)	296	17.9 (8.2)

### Relationship between MUAC and BMI

Complete data for MUAC and BMI were available for 303 patients (Fig 1); 211 male and 92 female, with 10% of each group over 65 years of age. Approximately 50–60% of these patients were 41–65 years old **(**Table 1).

**Fig 2:**
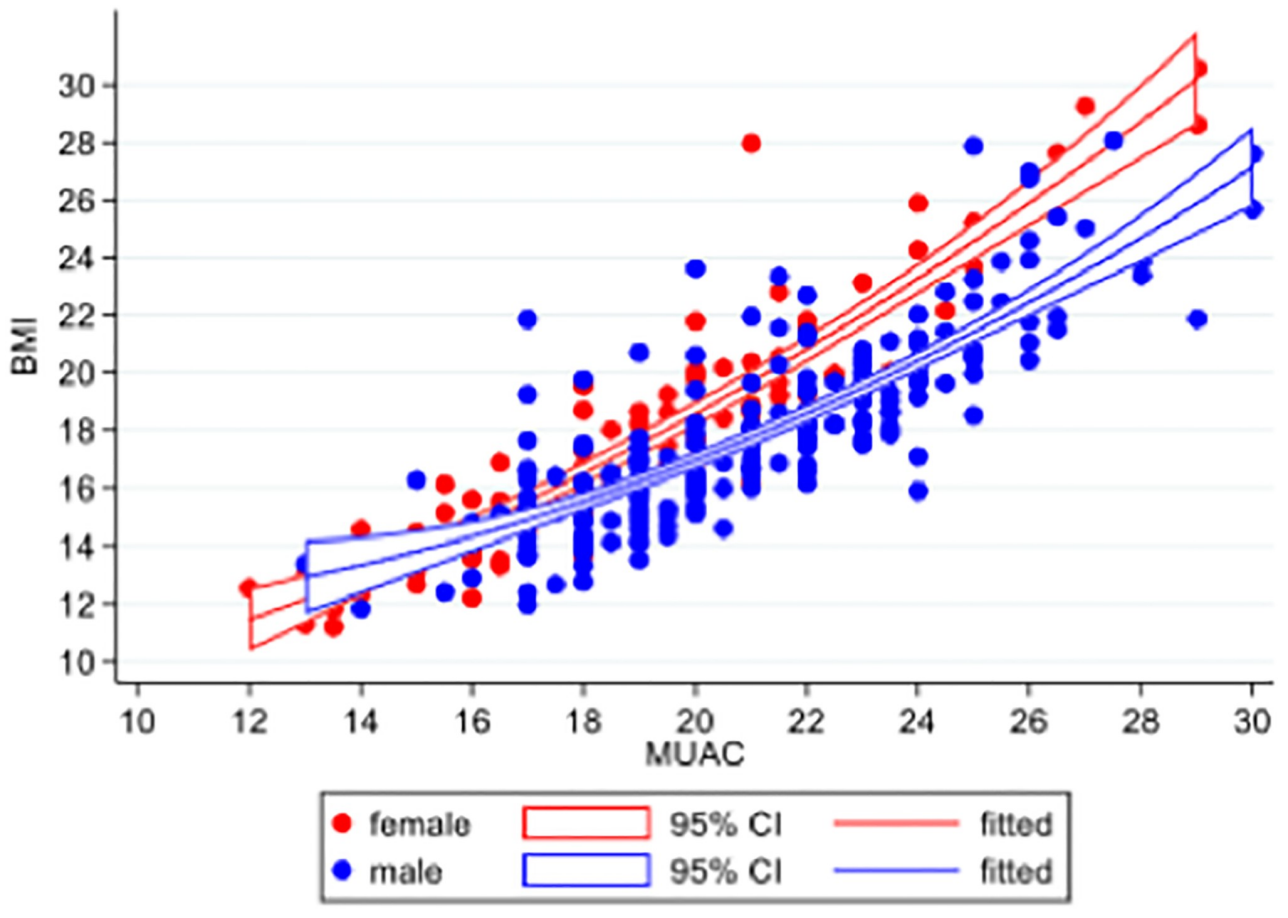
Relationship between MUAC and BMI, by sex (red = female, blue = male). Dots represent observed data. Lines are fitted values from quadratic relationship between MUAC and BMI, within each sex sub-group. The quadratic relationship was significant, as was an interaction between sex and MUAC.

We hypothesised *a priori* that the relationship between MUAC and BMI would be modified by sex. We also considered it plausible that the relationship between MUAC and BMI could vary by age: three categories 18–40, 41–65 & >65 years. Linear regression models were used to assess evidence of effect modification of the relationship between MUAC and BMI by age and sex, to determine whether stratification by age or sex would be needed, in subsequent analyses for identifying a suitable MUAC cut-off to classify a patient as malnourished based on their BMI value. The relationship between MUAC and BMI was quadratic. There was evidence of a significant interaction between the quadratic MUAC relationship with BMI and sex (LRT p<0.001) but not of an interaction between age group and MUAC (LRT p = 0.154), or a MUAC-sex-age group interaction (p = 0.114). Therefore, subsequent evaluation of MUAC performance to predict BMI<17 kg/m^2^ and BMI<16 kg/m^2^ was stratified by sex only. Observed and predicted values from linear regression modelling with a quadratic fit for MUAC are presented graphically by sex in (Fig 2).

### Evaluation of MUAC cut-off values to diagnose malnutrition

Different cut-off values for MUAC to diagnose moderate under-nutrition (BMI <17 kg/m^2^) were identified for male and female patients, based on the highest sensitivity (lowest risk of missed cases) for specificity of ≥80% (Table 2). The AUC was 0.96 for females and 0.92 for males (S1 Fig). Sensitivity was 91% for a cut-off of 18.5 cm for females and 89% for a cut-off of 20.5 cm for males (Table 2). In our population, this would have resulted in 15 (5%) missed diagnoses of under-nutrition based on BMI<17 kg/m^2^ and one missed diagnosis of severe under-nutrition with BMI<16 kg/m^2^. On the other hand, 23 individuals (7.6%) would have been falsely diagnosed as moderately malnourished (false positives) (S1 Table), of whom 10 would actually have had a normal BMI of ≥18.5 kg/m^2^.

**Table 2:** Evaluation of MUAC as a predictor of moderate acute under-nutrition defined as BMI<17 kg/m^2^.

MUAC cut-off	Sensitivity	Specificity	PPV	NPV	FNR
**Female:**					
17.5	0.69 (0.53–0.82)	1.00 (0.92–1.00)	1.00 (0.89–1.00)	0.77 (0.65–0.87)	0.31 (0.18–0.47)
18	0.89 (0.76–0.96)	0.91 (0.80–0.98)	0.91 (0.78–0.97)	0.90 (0.77–0.97)	0.11 (0.04–0.24)
**18.5**	**0.91 (0.79–0.98)**	**0.89 (0.77–0.96)**	**0.89 (0.76–0.96)**	**0.91 (0.79–0.98)**	**0.09 (0.02–0.21)**
19	0.91 (0.79–0.98)	0.83 (0.69–0.92)	0.84 (0.70–0.93)	0.91 (0.78–0.97)	0.09 (0.02–0.21)
19.5	0.93 (0.82–0.99)	0.77 (0.62–0.88)	0.79 (0.66–0.89)	0.92 (0.79–0.98)	0.07 (0.01–0.18)
20	0.98 (0.88–1.00)	0.68 (0.53–0.81)	0.75 (0.62–0.85)	0.97 (0.84–1.00)	0.02 (0.00–0.12)
20.5	0.98 (0.88–1.00)	0.64 (0.49–0.77)	0.72 (0.59–0.83)	0.97 (0.83–1.00)	0.02 (0.00–0.12)
21	1.00 (0.92–1.00)	0.53 (0.38–0.68)	0.67 (0.55–0.78)	1.00 (0.86–1.00)	0.00 (0.00–0.08)
**Male:**					
19	0.70 (0.60–0.79)	0.90 (0.84–0.95)	0.86 (0.76–0.93)	0.78 (0.70–0.85)	0.30 (0.21–0.40)
19.5	0.75 (0.65–0.83)	0.90 (0.82–0.94)	0.86 (0.76–0.92)	0.81 (0.73–0.88)	0.25 (0.17–0.35)
20	0.85 (0.77–0.92)	0.84 (0.76–0.90)	0.82 (0.73–0.89)	0.87 (0.80–0.93)	0.15 (0.08–0.23)
**20.5**	**0.89 (0.80–0.94)**	**0.84 (0.76–0.90)**	**0.83 (0.74–0.89)**	**0.90 (0.83–0.95)**	**0.11 (0.06–0.20)**
21	0.95 (0.88–0.98)	0.74 (0.65–0.82)	0.75 (0.67–0.83)	0.94 (0.88–0.98)	0.05 (0.02–0.12)
21.5	0.96 (0.90–0.99)	0.70 (0.60–0.78)	0.72 (0.64–0.80)	0.95 (0.88–0.99)	0.04 (0.01–0.10)
22	0.99 (0.94–1.00)	0.57 (0.47–0.66)	0.66 (0.57–0.73)	0.98 (0.92–1.00)	0.01 (0.00–0.06)

Data are proportions (95% CI). PPV = positive predictive value, NPV = negative predictive value, FNR = false negative rate

For severe under-nutrition based on BMI<16 kg/m^2^, (Table 3) the best performing cut-off for females was 18 cm based on highest sensitivity (100%) for specificity ≥80% and 19.5 cm for males (sensitivity: 89%). Using 18.5cm for BMI<17 kg/m^2^ for females, applied to severe under-nutrition as BMI<16 kg/m^2^, led to negligible difference in performance measures compared to the lower cut off of 18cm for under-nutrition defined as BMI<16 kg/m^2^. In contrast, using the same cut-off for males for under-nutrition defined as BMI<17 kg/m^2^ and BMI<16 kg/m^2^ resulted in inadequate specificity (<80%, Table 3). If we employed the lower 19.5 cm MUAC cut off for a BMI<16 kg/m^2^, it would have resulted in 7 (2.3%) false negatives (missed cases) but 35 (11.6%) false positives (S1 Table).

**Table 3:** Evaluation of MUAC as a predictor of severe acute under-nutrition defined as BMI<16 kg/m^2^. Data are proportions (95% CI). PPV = positive predictive value, NPV = negative predictove value, FNR = false negative rate.

MUAC cut-off	Sensitivity	Specificity	PPV	NPV	FNR
**Female:**					
17	0.81 (0.64–0.92)	0.96 (0.88–1.00)	0.94 (0.79–0.99)	0.89 (0.78–0.95)	0.19 (0.08–0.36)
17.5	0.81 (0.64–0.92)	0.96 (0.88–1.00)	0.94 (0.79–0.99)	0.89 (0.78–0.95)	0.19 (0.08–0.36)
18	**1.00 (0.90–1.00)**	**0.86 (0.74–0.94)**	**0.82 (0.67–0.92)**	**1.00 (0.93–1.00)**	**0.00 (0.00–0.10)**
18.5	1.00 (0.90–1.00)	0.82 (0.70–0.91)	0.78 (0.64–0.89)	1.00 (0.92–1.00)	0.00 (0.00–0.10)
19	1.00 (0.90–1.00)	0.77 (0.64–0.87)	0.73 (0.59–0.85)	1.00 (0.92–1.00)	0.00 (0.00–0.10)
19.5	1.00 (0.90–1.00)	0.70 (0.56–0.81)	0.68 (0.54–0.80)	1.00 (0.91–1.00)	0.00 (0.00–0.10)
**Male:**					
18.5	0.64 (0.51–0.76)	0.89 (0.83–0.94)	0.72 (0.58–0.83)	0.85 (0.78–0.90)	0.36 (0.24–0.49)
19	0.81 (0.70–0.90)	0.82 (0.75–0.88)	0.67 (0.55–0.77)	0.91 (0.85–0.95)	0.19 (0.10–0.30)
19.5	**0.89 (0.79–0.95)**	**0.82 (0.74–0.88)**	**0.68 (0.57–0.78)**	**0.94 (0.89–0.98)**	**0.11 (0.05–0.21)**
20	0.95 (0.87–0.99)	0.73 (0.66–0.80)	0.61 (0.51–0.71)	0.97 (0.92–0.99)	0.05 (0.01–0.13)
20.5	0.98 (0.92–1.00)	0.73 (0.65–0.80)	0.61 (0.51–0.71)	0.99 (0.95–1.00)	0.02 (0.00–0.08)
21	0.98 (0.92–1.00)	0.61 (0.52–0.68)	0.52 (0.43–0.61)	0.99 (0.94–1.00)	0.02 (0.00–0.08)

### Limb length as a predictor of height

**Fig 3:**
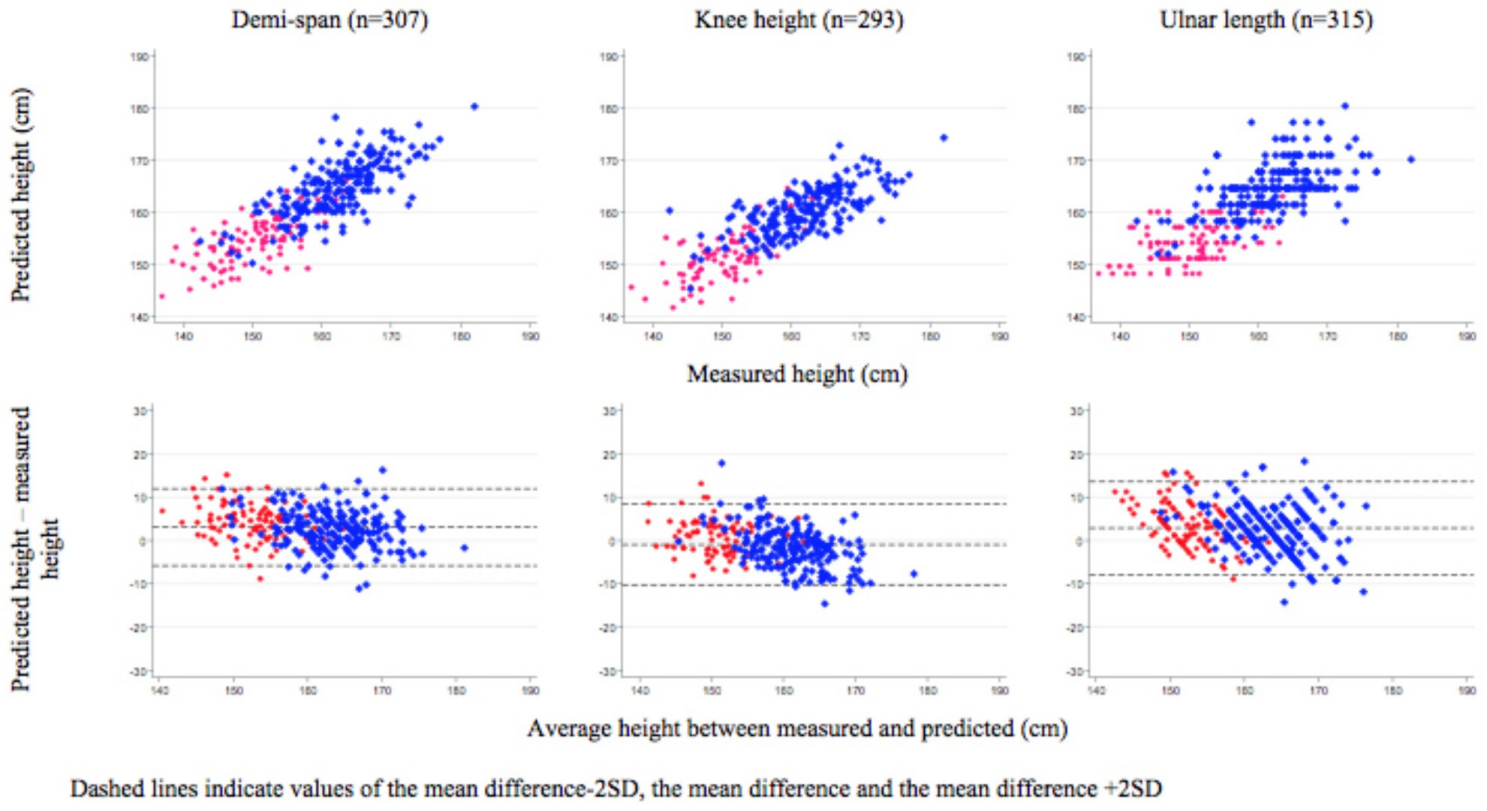
Predicted height by limb length and sex compared to measured height.

The number of patients included in each limb length analysis to predict height and then BMI are shown in Fig 1. The mean difference between predicted and observed heights (95% CI) was 3.09 cm (2.59, 3.59) for demi-span, -0.98 cm (-1.51, -0.44) for knee height, and 2.85 cm (2.26, 3.45) for ulnar length (Fig 3). The Bland-Altman plot for knee height suggested that the mean difference in predicted-measured height tended to decrease as true height increased, which was not observed for demi-span or ulnar length.

### Using predicted height values to calculate BMI and classify nutritional status

All three limb lengths had high sensitivity and specificity for accurately detecting both moderate severe malnutrition, achieving a minimum specificity of 84% with a corresponding maximum sensitivity of 96–97% (S2 Table). Using demi-span resulted in the lowest proportion of missed cases (9/294, 3.1%) out of the three limb length measures with none classified as BMI≥17 kg/m^2^ when measured BMI was less than 16 kg/m^2^.

### Relationship between handgrip strength, percentage body fat and BMI

**Fig 4:**
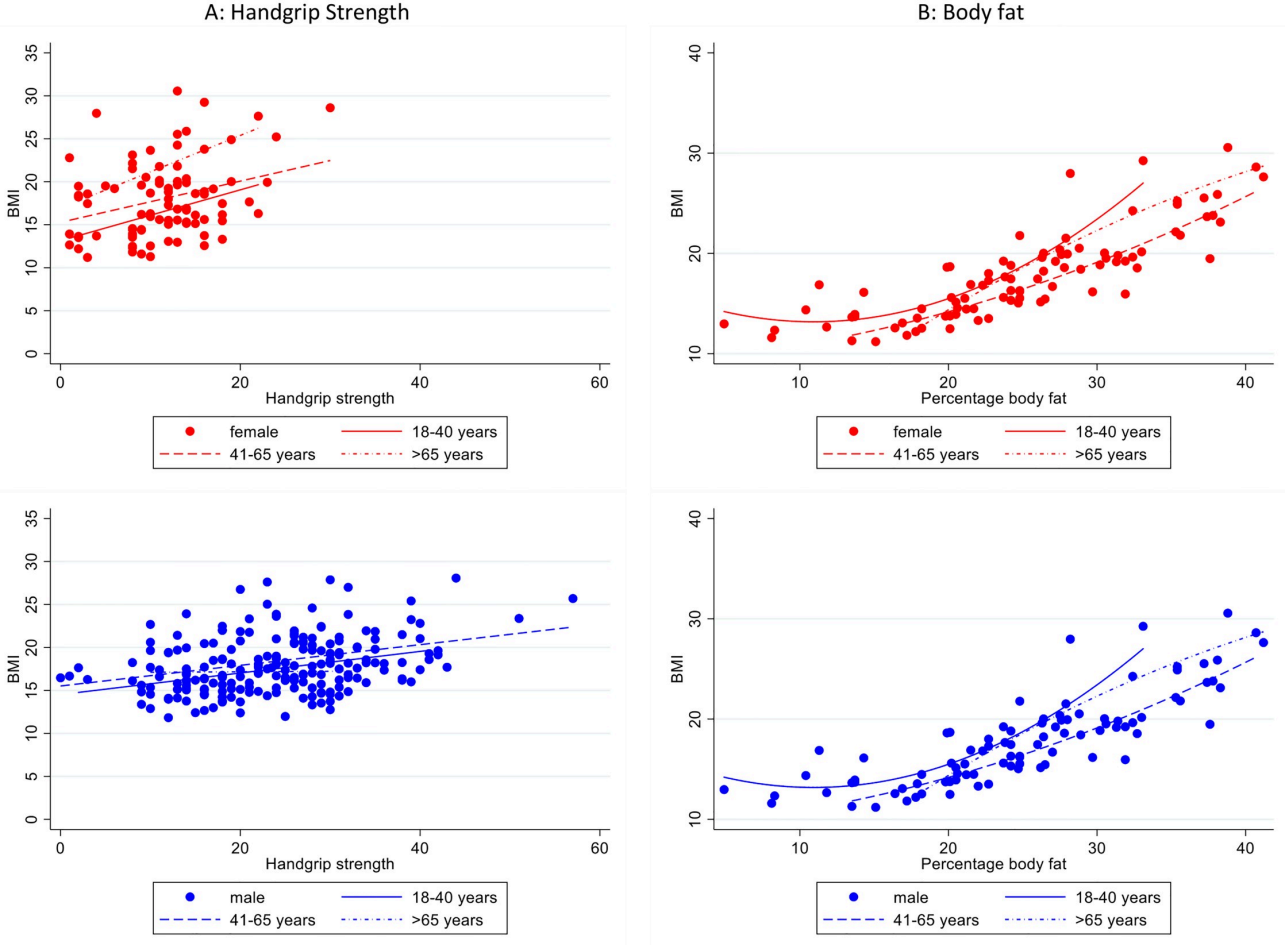
Relationship between body fat, handgrip strength and BMI, by sex (red = female, blue = male) and age group. Lines are fitted values from a linear relationship between handgrip strength and BMI and a quadratic relationship between percentage body fat and BMI, within each sex and age sub-group.

To visualise the relationship between each of handgrip strength and percentage body fat and BMI, we followed the same analysis approach for MUAC and BMI modelled as continuous variables. The quadratic term for handgrip strength was not significant (LRT p = 0.240). There was some evidence of a sex-handgrip interaction (LRT p = 0.040), but not for an age group-handgrip interaction (LRT p = 0.126), and for a sex-age-handgrip interaction (LRT p = 0.040), after accounting for the handgrip-sex interaction (Fig 4). If cut-off values for grip-strength to predict BMI<17kg/m^2^ were to be explored, age and sex specific cut-offs may need to be explored.

There was a quadratic relationship between percentage body fat and BMI (LRT p = 0.020 for quadratic term). Although there was no evidence of a sex-body fat percentage interaction (LRT p = 0.153) and some evidence of an age group-body fat interaction (LRT p = 0.012), there was evidence of a three-way interaction between sex, age group and body fat percentage (LRT p = 0.005, Fig 4).

Out of the three measures (MUAC, percentage body fat and hand grip strength), the regression model for MUAC was the simplest, in terms of only having one interaction with sex. In addition, of the final models illustrated in the graphs in Figs 2 and 4, the model for MUAC had the highest adjusted R^2^ value (MUAC R^2^ = 0.77, handgrip strength R^2^ = 0.14, body fat percentage R^2^ = 0.66).

## Discussion

This study evaluated alternative methods to reliably diagnose moderate and severe under-nutrition in an acutely unwell Filipino in-patient population. Among the study population typical diagnosis based on BMI were not possible for a substantial proportion of patients. Patients who could not be diagnosed using BMI likely represent those with advanced clinical disease and were therefore at a greater risk of malnutrition and poor clinical outcome.

The MUAC cut-off values we identified performed well, compared to diagnosis based on BMI in patients who had both BMI and MUAC data available. Of the 45 patients with MUAC but not BMI, 35 (78%) had MUAC less than our proposed cut-offs suggesting that use of MUAC compared to BMI may identify a further 10% of patients as having moderate or severe under-nutrition. Under similar conditions of a moderate or high prevalence of moderate/severe under-nutrition for both sexes, these cut-offs result in high positive and negative predictive values on which clinicians can make patient level decisions regarding treatment. A MUAC based diagnostic method is simple and quick to implement, once adequate training is provided. However, there was limited ability to differentiate between moderate (BMI<17 kg/m^2^) and severe under-nutrition as defined by BMI<16 kg/m^2^, especially in women. This has implications where different types of intervention may be proposed for severe compared to moderate under-nutrition. Current efforts to determine MUAC cut-offs for adults have primarily focused on a cut-off correlating with BMI of <18.5kg/m^2^, [11] but there are no globally agreed cut-offs for adults for any adult BMI or for any health related-outcome, despite some country/agency specific cut-offs for pregnant women, HIV patients and for entry into feeding programmes, mostly in emergency situations. As TB related nutrition interventions would likely be offered targeted to those with moderate/severe underweight, we focused on a cut-off of <17kg/m^2^. To our knowledge this is the first published report to determine the performance of different MUAC cut-offs to predict a BMI of 17.0 kg/m^2^. Our proposed cut-offs are similar compared to previous MUAC cut-offs of 16.1–18.5 cm for both sexes (in the absence of complications, including bilateral pitting oedema) for recommendation for admission to adult supplementary feeding in acute famine situations [28] and more recent country-specific (all in Africa) cut offs used for HIV care and management of adult malnutrition programmes of mostly between18-19 cm for severe under-nutrition and mostly between 21.0 to 23.0 cm range for moderate under-nutrition, but with no difference by sex [29] [30].

Where weight can be obtained but not height, it is possible to obtain reasonable predicted height based on limb lengths using established equations which can then be used to calculate BMI with similar or slightly better predictive performance than use of MUAC. However, this approach requires several steps and a number of calculations based on equations that are not straight forward. Implementation would likely require development of a tool or chart to obtain heights from limb lengths and would still result in possibilities for measurement error at two stages; predicting height and calculating BMI. Although knee height resulted in the smallest difference between mean predicted and measured height, fewer measurements were available, indicating the increased difficulty in obtaining this measurement compared to demi-span or ulnar length, both of which over-estimated height in this population and therefore had the highest sensitivity, but lower specificity in BMI calculations than knee height, for which predicted height also varied with increasing true height.

Body fat percentage calculated using a standard equation by skilled trained staff was strongly associated with BMI, handgrip strength less so. For both of these measurements, the possibility of differing relationships with BMI by age and sex, combined with more complex measurement process do not provide a strong case for their use in diagnosing under-nutrition, over a simpler method such as MUAC. However, it is not yet known how these measures relate to risk of adverse outcomes. Grip-strength is an indicator of muscle mass and function and in catabolic conditions like TB may be more indicative of risk than either MUAC or BMI which encompass assessment of both lean and fat mass and predicted all-cause mortality in a multi-country study [31].

### Strengths and limitations

A strength of this study is the relatively large sample size with a range of ages with accurate measurements conducted under research conditions by skilled staff. As shown by the datasets in a recent, large individual participant data meta-analysis conducted by Tang and colleagues, which assessed the performance of different MUAC cut-offs for a BMI of <18.5 [11], our dataset with a wide range of lower BMI values and high proportion of under-nutrition and greater proportion of men, is rare. Unlike the results of this meta-analysis, our data strongly support the requirement for sex-specific MUAC cut-offs, this may relate to the greater proportion of males in our dataset and/or sex-specific effects of TB on body composition. Although HIV status was unknown for a large proportion of patients, the prevalence of HIV co-infection in Filipino TB patients is low compared to most African countries [32]. In resource-limited settings a cut-off for moderate under-nutrition may be especially important to allow identification of those who should be given the highest priority for interventions, particularly in TB programmes, in which very high proportions may have mild malnutrition, at least at initiation of treatment.

One limitation of the study is that we were not able to obtain measured BMI on all patients in the larger cohort which reduced our sample size, whilst the stratification by sex in the primary sensitivity/specificity analysis of MUAC for predicting BMI <17 kg/m^2^ reduced it further. However, the results were still within our expected level of precision. However, this reinforces the point that alternative methods of estimating BMI and therefore nutritional risk are needed in this population. Another limitation is that, BMI is affected by body shape and we did not assess sitting height to be able to correct BMI for the cormic index (sitting height to standing height ratio) [28], nor could we find any data on the cormic index in Filipino populations to estimate what, if any, effect this might have on our proportions classified as moderate and severely under-nourished [33]. Also, we did not systematically assess for odema or bilateral pitting odema by our research nurses, but relied on extraction from the clinical records, and thus undiagnosed odema might also have affected BMI. Additionally, caution should be exercised in extrapolating these results to other populations with similar levels of under-nutrition, but of different aetiology, for example starvation compared to disease-induced catabolic processes, which could alter the nature of the relationship of MUAC (and also body fat percentage, and grip-strength) with BMI, in particular how MUAC relates to BMI by age and sex.

The use of MUAC as a simple accessible screening tool for under-nutrition in adults, in either inpatient, outpatient and community settings has great potential for facilitating the documentation, diagnosis, prevention and treatment of adult malnutrition, an often ignored co-morbidity, especially in tuberculosis patients. Furthermore, although evidence is available from very few studies in adults, MUAC may be better than BMI for predicting risk of adverse outcomes including death. For example in adults with very severe acute malnutrition in a famine setting, [34]; in adult acute admissions to London Hospitals [35] and deaths during TB treatment in adult HIV positive patients under demographic surveillance in Guinea Bissau [36]. Finally, it is likely that MUAC may be more responsive in detecting changes in nutritional status as a result of therapeutic feeding or intervention and therefore represent a simple tool for patient monitoring compared to repeated weight measurements, but little published data is currently available [30].

### Conclusions

Sex-specific MUAC cut-off values are proposed that offer a cheap and simple diagnostic tool for moderate/severe under-nutrition in acutely unwell adults presenting with TB disease in hospital settings. This diagnostic method should be particularly useful for timely identification of patients in most need of nutritional interventions and in patients who are too immobile to have height and weight measurements taken for a BMI based diagnosis. Results of this study provide opportunities for implementation of MUAC based diagnosis of under-nutrition and evaluations of the usefulness of MUAC based diagnosis in other adult-based clinical settings Further studies are required to determine if these same cut-offs can be applied to populations of different ethnic background and with different underlying causes of under-nutrition. Furthermore, more research is required to determine the usefulness of MUAC for monitoring responsiveness to therapeutic interventions, and in investigations of links between under-nutrition and important clinical endpoints such as TB treatment failure and death in TB programmes.

## Supporting information

S1 File Durnin and Wormersley equation for calculating percentage body fat. (DOCX)

S2 File STARD checklist. (DOCX)

S1 Table Classification of BMI defined malnutrition by MUAC: [A] moderate or severe, BMI<17 kg/m^2^; [B] severe, BMI <16.0 kg/m^2^. (DOCX)

S2 Table Performance of BMI from predicted height to accurately classify under-nutrition grade using BMI from measured height. (DOCX)

S1 Fig Receiver operating curves for MUAC as a predictor of under-nutrition defined by BMI <17 kg/m^2^ or <16 kg/m^2^. (DOCX)
