# Supplementary material for: Performance of alternative measures to body mass index in the assessment of moderate and severe under-nutrition among acutely unwell patients hospitalized in a TB ward in the Philippines: A cross-sectional study
Source: PLoS One. 2019 May 16;14(5):e0215968. doi: 10.1371/journal.pone.0215968 (PMC6522031; doi:10.1371/journal.pone.0215968)
Supplement: S1 File — (DOCX) [file pone.0215968.s001.docx]

**S1 File. Durnin and Wormersley Equation for calculating percentage body fat.**

| Age | Males | Females |
| --- | --- | --- |
| 17-19 | D = 1.1620 - (0.0630 X L) | D = 1.1549 - (0.0678 X L) |
| 20-29 | D = 1.1631 - (0.0632 X L) | D = 1.1599 - (0.0717 X L) |
| 30-39 | D = 1.1422 - (0.0544 X L) | D = 1.1423 - (0.0632 X L) |
| 40 -49 | D = 1.1620 - (0.0700 X L) | D = 1.1333 - (0.0612 X L) |
| > 50 | D = 1.1715 - (0.0779 X L) | D = 1.1339 - (0.0645 X L) |

D = the predicted density of the body (g/ml)

L = log10 of the sum of (triceps + biceps + subscapular + asuprailiac skinfolds in mm)

Siri equation to convert D to percentage body fat = (495/D)-450
