# Supplementary material for: Performance of alternative measures to body mass index in the assessment of moderate and severe under-nutrition among acutely unwell patients hospitalized in a TB ward in the Philippines: A cross-sectional study
Source: PLoS One. 2019 May 16;14(5):e0215968. doi: 10.1371/journal.pone.0215968 (PMC6522031; doi:10.1371/journal.pone.0215968)
Supplement: S1 Table — (DOCX) [file pone.0215968.s003.docx]

**S1 Table. Classification of BMI defined malnutrition by MUAC: [A] moderate or severe, BMI<17 kg/m^2^; [B] severe, BMI <16.0 kg/m^2^.**

**S1A Table**

| BMI<17 | BMI < 17 predicted by MUAC (<18.5 female) or <20.5 (male) | |  |
| --- | --- | --- | --- |
|  | No | Yes | Total |
| No | **139** | 23 | 162 |
| Yes | 15 | **126** | 141 |
| Total | 154 | 149 | 303 |

**S1B Table**

| BMI<16 | BMI < 16 predicted by MUAC (<18.0 female) or <19.5 (male) | |  |
| --- | --- | --- | --- |
|  | No | Yes | Total |
| No | **168** | 35 | 203 |
| Yes | 7 | **93** | 100 |
| Total | 175 | 128 | 303 |
