# Supplementary material for: Performance of alternative measures to body mass index in the assessment of moderate and severe under-nutrition among acutely unwell patients hospitalized in a TB ward in the Philippines: A cross-sectional study
Source: PLoS One. 2019 May 16;14(5):e0215968. doi: 10.1371/journal.pone.0215968 (PMC6522031; doi:10.1371/journal.pone.0215968)
Supplement: S2 Table — (DOCX) [file pone.0215968.s004.docx]

**S2 Table. Performance of BMI from predicted height to accurately classify under-nutrition grade using BMI from measured height.**

* BMI (kg/m^2^) cut-off value for moderate or severe malnutrition

| Height predictor | BMI* | N | Sensitivity | Specificity | PPV | NPV | FNR | AUC |
| --- | --- | --- | --- | --- | --- | --- | --- | --- |
| Demi | 17 | 294 | 0.96 (0.91 - 0.98) | 0.84 (0.77 - 0.89) | 0.84 (0.77 - 0.89) | 0.96 (0.91 - 0.98) | 0.04 (0.02 - 0.09) | 0.80 (0.74 - 0.86) |
| Knee | 17 | 284 | 0.88 (0.82 - 0.93) | 0.96 (0.92 - 0.99) | 0.95 (0.90 - 0.98) | 0.91 (0.85 - 0.95) | 0.12 (0.07 - 0.18) | 0.85 (0.79 - 0.91) |
| Ulnar | 17 | 302 | **0.96 (0.91 - 0.98)** | **0.89 (0.83 - 0.93)** | **0.88 (0.82 - 0.93)** | **0.96 (0.91 - 0.99)** | **0.04 (0.02 - 0.09)** | **0.85 (0.80 - 0.91)** |
| Demi | 16 | 294 | **0.97 (0.91 - 0.99)** | **0.84 (0.78 - 0.89)** | **0.76 (0.67 - 0.83)** | **0.98 (0.95 - 1.00)** | **0.03 (0.01 - 0.09)** | **0.82 (0.76 - 0.87)** |
| Knee | 16 | 284 | 0.86 (0.77 - 0.92) | 0.94 (0.90 - 0.97) | 0.88 (0.79 - 0.94) | 0.93 (0.89 - 0.96) | 0.14 (0.08 - 0.23) | 0.81 (0.73 - 0.88) |
| Ulnar | 16 | 302 | 0.95 (0.89 - 0.98) | 0.86 (0.80 - 0.90) | 0.77 (0.68 - 0.84) | 0.97 (0.94 - 0.99) | 0.05 (0.02 - 0.11) | 0.81 (0.75 - 0.87) |
