# Supplementary material for: Performance of alternative measures to body mass index in the assessment of moderate and severe under-nutrition among acutely unwell patients hospitalized in a TB ward in the Philippines: A cross-sectional study
Source: PLoS One. 2019 May 16;14(5):e0215968. doi: 10.1371/journal.pone.0215968 (PMC6522031; doi:10.1371/journal.pone.0215968)
Supplement: S1 Fig — (DOCX) [file pone.0215968.s005.docx]

**S1 Fig. Receiver operating curves for MUAC as a predictor of under-nutrition defined by BMI <17 kg/m^2^ or <16 kg/m^2^**

| BMI <17 kg/m^2^ | All:   | Female:   | Male:   |
| --- | --- | --- | --- |
| BMI <16 kg/m^2^ |  |  |  |
